# Comparison of Pharmacological Therapies in Relapse Rates in Patients With Relapsing-Remitting Multiple Sclerosis

**DOI:** 10.7759/cureus.45454

**Published:** 2023-09-18

**Authors:** Indu Etta, Ruaa Elballushi, Viktoriia Kolesnyk, Kim P Sia, Sana Rehman, Sehrish Arif, Sania J Moonnumackel, Arun Nair

**Affiliations:** 1 Department of Internal Medicine, Kakatiya Medical College, Warangal, IND; 2 School of Medicine, Royal College of Surgeons in Ireland - Medical University of Bahrain, Muharraq, BHR; 3 First Faculty of Medicine, Charles University, Prague, CZE; 4 School of Medicine, Emilio Aguinaldo College, Manila, PHL; 5 College of Medicine, Fatima Memorial Hospital (FMH) College of Medicine and Dentistry, Lahore, PAK; 6 Department of Internal Medicine, Medical City Fort Worth, Fort Worth, USA; 7 Department of Pediatrics, Saint Peter’s University Hospital, Somerset, USA

**Keywords:** types of multiple sclerosis, multiple sclerosis, relapse rates, pharmacotherapy, relapsing-remitting multiple sclerosis

## Abstract

Multiple sclerosis (MS) is a chronic autoimmune neurological disorder that significantly impacts the central nervous system (CNS), which includes the brain and spinal cord. Approximately 2.8 million individuals are believed to be living with MS worldwide. The management of MS has evolved considerably over the years, offering a multitude of guidelines, diverse treatment options, and different approaches to signs and symptoms. The present systematic literature review serves as a comprehensive analysis of the current therapeutic options for MS. It provides a thorough literature review of Food and Drug Administration (FDA)-approved drugs comparing their various clinical end points while concurrently assessing their risk-benefit ratio. It also provides an extensive review of current guidelines and offers an in-depth examination of the different approaches to MS. Through this multifaceted approach, this paper facilitates easy access to available treatment options and aims to aid healthcare providers in decision-making as well as providing a foundation for future research aimed at enhancing treatment options for MS.

## Introduction and background

Multiple sclerosis (MS) is a central nervous system (CNS) autoimmune disorder characterized by inflammation, demyelination, and neuroaxonal degeneration. Oxidative stress is one of the contributing factors to disease development [1]. An unknown antigen triggers the immune response (Th1 and Th17 cells) that produces pro-inflammatory cytokines. These cytokines produce metalloproteinases that disintegrate the blood-brain barrier (BBB). Thus, inflammatory cells cross the BBB and lead to inflammation [2]. Clinically, multiple sclerosis is classified into four types: relapsing-remitting MS (RRMS), primary progressive MS, secondary progressive MS (SPMS), and relapsing progressive MS. Roughly 85% of patients with multiple sclerosis develop relapsing-remitting type at the onset and gradually become worse with less frequent relapses (secondary progressive MS). Less than 10% of patients directly show progressive symptoms and neurological deficits (primary progressive MS) [3]. Due to different hormonal changes, the prevalence of MS differs significantly between males and females; incidence between genders also differs between different types of MS [4]. Relapsing MS is more predominant in females than in males, while primary progressive MS occurs at equal rates in both males and females [3,4].

The diagnosis of multiple sclerosis is based on the presence of magnetic resonance imaging (MRI) lesions in MS-typical regions of CNS such as periventricular, juxtacortical, and infratentorial, and CSF abnormalities such as mononuclear cell pleocytosis and increased IgG [3]. Clinical syndromes typical for multiple sclerosis are unilateral optic neuritis, diplopia (due to internuclear ophthalmoplegia), facial sensory loss or trigeminal neuralgia, cerebellar syndromes (ataxia and nystagmus), sensory impairment, or motor weakness. The development of effective therapies, such as disease-modifying therapies (DMTs), for multiple sclerosis are specified pharmacological treatments that are specifically focused on controlling relapse rates and decreasing disease progression and disabilities. Treatment guidelines for multiple sclerosis have been published by different associations such as the Association of British Neurologists (ABN), the American Academy of Neurology (AAN), the European Committee on Treatment and Research in Multiple Sclerosis working jointly with the European Academy of Neurology (ECTRIMS/EAN), and the Brazilian Academy of Neurology working jointly with Brazilian Committee on Treatment and Research in Multiple Sclerosis (BAN/BCTRIMS) [5].

The first approved therapies for MS by the Food and Drug Administration (FDA) are interferons and glatiramer acetate [3]. Later, on knowing the pathogenesis of MS, newer therapies have emerged, such as lymphocyte adhesion blockers (natalizumab), sphingosine-1-phosphate (S1P) receptor modulators (fingolimod (FTY), siponimod, and ozanimod), anti-CD20 monoclonal antibody (rituximab, ocrelizumab, and ofatumumab), nuclear factor-like 2 pathway inhibitor (dimethyl fumarate (DMF) and diroximel fumarate (DRF)), and dihydroorotate dehydrogenase inhibitor (teriflunomide) [3]. Other infrequently used DMTs are cladribine, mitoxantrone, and alemtuzumab. Bruton tyrosine kinase (BTK) inhibitors (tolebrutinib) are non-DMTs used for MS that are currently being investigated.

The diagnosis of MS is mostly made in the relapsing-remitting form of MS. Thus, choosing an effective treatment at this stage is crucial to minimize relapse rates and alleviate the symptoms of MS. With the increased advancements in the treatment for MS, there is a wide variety of drugs with variation in relapse rates and period of remission between attacks, whose comparison is the prime target of this study.

There have been many studies conducted to establish the efficacy and safety of DMTs. All existing studies show the comparison between a few drugs as it is not feasible to compare all the available DMTs in a research setting. This study aims to enhance our understanding and decision-making in multiple sclerosis treatment. It includes a systematic literature review comparing different clinical end points of available drugs such as relapse rates and assessing the risk-benefit ratio of therapeutic options. Additionally, this study evaluates the validity of old, current, and emerging treatment guidelines. This study also provides a comprehensive literature review of FDA-approved drugs for MS and establishes a systematic organization of these drugs for easy access and future reference.

## Review

The current standard of treatment for relapsing-remitting multiple sclerosis

Disease-modifying therapies (DMTs) have been developed to modify the course of MS by reducing relapse rates and slowing the progression of the disease. They were invented to reduce the frequency and severity of relapses and ultimately delay disability. DMTs work by targeting the immune system by modulating, altering, neutralizing, or changing the course of action of some of the body’s immune processes to pathology. Choosing the right DMTs depends on the character of MS, type, and severity, as well as the patient’s demographic, age, and comorbidity. Since DMTs work on the immune system, adverse effects (AEs) and the lack of proper response by the immune system remain a constant limitation to a lot of DMTs in the market. More effective and safer therapies are needed to overcome some of the current limitations and develop personalized treatment options.

Table 1 categorizes MS drug treatments based on their FDA approval status. It distinguishes between drugs approved before and after 2015, thereby enabling a reliable reference for healthcare professionals regarding old and relatively new treatments available in the current market. The table also lists drugs pending FDA approval and those that were discontinued by the FDA to allow for a comprehensive overview.

**Table 1:** FDA-approved drugs for multiple sclerosis FDA: Food and Drug Administration, RRMS: relapsing-remitting multiple sclerosis, S1P: sphingosine-1-phosphate Source: [6]

Name of drug		Date of approval
Drugs approved prior to 2015		
Ampyra - dalfampridine	2010	
Aubagio - teriflunomide	2012	
Avonex - interferon beta-1a	1996	
Copaxone - glatiramer acetate injections	1997	
Extavia - interferon beta-1b	2009	
Gilenya - fingolimod S1P modulator	2010	
Lemtrada - alemtuzumab	2014	
Plegridy - peginterferon beta-1a	2014	
Rebif - interferon beta-1a	2002	
Tecfidera - dimethyl fumarate	2013	
Tysabri - natalizumab	2004	
Drugs approved after 2015		
Kesimpta - ofatumumab	2020	
Mavenclad - cladribine	2019	
Mayzent - siponimod	2019	
Ocrevus - ocrelizumab	2017	
Ponvory - ponesimod	2021	
Vumerity - diroximel fumarate	2019	
Zeposia - ozanimod	2020	
Bafiertam - monomethyl fumarate	2020	
Drugs in clinical trials (pending FDA approval)		
Amiselimod	Currently phase II clinical trial for RRMS	
GSK2018682	Successfully finished phase II clinical trial	
Discontinued drugs		
Novantrone - mitoxantrone	Approved by the FDA in 2000, discontinued clinical use because of adverse effects	
Ceralifimod	Discontinued after phase II	

DMTs are classified into first-line, second-line, and third-line treatments (Table 2), based on their clinical end points in regard to their safety, efficacy, sensitivity, specificity, relapse rates, morbidity, and mortality. Table 3 and Table 4 provide a systematic review of existing and new treatments for MS by exploring their relevant clinical end points as well as the mechanism of action and possible adverse effects.

**Table 2:** List of first-, second-, and third-line approved therapies for RRMS RRMS: relapsing-remitting multiple sclerosis Source: [7]

First line	Second line	Third line
Interferon beta	Natalizumab	Mitoxantrone
Glatiramer	Fingolimod	Alemtuzumab
Teriflunomide	Azathioprine	Ocrelizumab
Dimethyl fumarate	Cladribine	

**Table 3:** Current versus established treatment for relapsing-remitting multiple sclerosis ARR: annualized relapse rate, MHC: major histocompatibility complex, NrF2: nuclear factor erythroid-derived 2-related factor, S1P: sphingosine-1-phosphate, S1P1: sphingosine-1-phosphate receptor subtype 1

Name of medication	Main mechanism of action	First line/second line/third line	Time from the onset of treatment to first relapses	Number of relapses during treatment course
Natalizumab [8]	Humanized monoclonal antibody against cell adhesion molecule α4-integrin	Second line	180-210 days	1
Fingolimod [9]	Oral S1P receptor modulator	Second line	720 days	ARR: 0.12 at up to 24 months
Interferon beta-1a [9]	Directly increase expression and concentration of anti-inflammatory agents while downregulating the expression of pro-inflammatory cytokines	First line	488 days	ARR: 0.67 at up to 24 months
Interferon beta [10]	Downregulation of the MHC class II expression present on antigen-presenting cells	First line	Not specified	Relapse rate is less in the interferon beta (0.84) compared to the placebo (1.27)
Dimethyl fumarate [11]	Reduces inflammation by activating the NrF2 pathway	First line	Not specified	One-year relapse rate: 0.028, two-year relapse rate: 0.071, time to relapse since treatment initiation: 0.957
Cladribine [5]	Purine antimetabolite	Second line	Not specified	0.14 and 0.15 for the 3.5 and 5.25 mg/kg doses, respectively
Ocrelizumab [5]	Humanized monoclonal anti-CD20 antibody	Third line	Not specified	ARR is lower in patients treated with ocrelizumab than in patients treated with interferon beta-1a (0.16 versus 0.29, p<0.001)
Ofatumumab [5]	Humanized monoclonal anti-CD20 antibody	Second line	Not specified	ARR is lower in patients treated with ofatumumab than in patients treated with teriflunomide (ASCLEPIOS I: 0.11 versus 0.22, ASCLEPIOS II: 0.10 versus 0.25, both p<0.001)
Ozanimod [5]	S1P1 modulator	Second line	Not specified	Significant reduction in relapses compared to interferon beta-1a
Ponesimod, siponimod, ozanimod, ceralifimod, GSK2018682, and amiselimod [12]	S1P receptor modulators: bioactive phospholipid regulating a range of cellular processes, including immunity, inflammation, angiogenesis, heart rate, smooth muscle tone, cell differentiation, cell migration and survival, calcium homeostasis, and endothelium integrity.	Second line	Within 180 days	
Newer disease-modifying therapies (dimethyl fumarate, fingolimod, teriflunomide, natalizumab, ocrelizumab, and alemtuzumab) [13]	Dimethyl fumarate - reduces inflammation by activating the NrF2pathway, fingolimod - oral S1P receptor modulator, teriflunomide - selectively and reversibly inhibits dihydro-orotate dehydrogenase, natalizumab - humanized monoclonal antibody against cell adhesion molecule α4-integrin, ocrelizumab - humanized monoclonal anti-CD20 antibody, alemtuzumab - monoclonal antibody, selectively binds to CD52, which is highly expressed on lymphocytes, depleting T and B cells from circulation in the periphery	Second and third line		Clinical relapses occurred in 53/103 (51%) patients at two years from treatment initiation

**Table 4:** Description of medications used in RRMS and associated adverse effects RRMS: relapsing-remitting multiple sclerosis, S1P: sphingosine-1-phosphate, HSV: herpes simplex virus, VZV: varicella-zoster virus, HTN: hypertension

Name of medication	Adverse effects
S1P modulators (fingolimod, ponesimod (Ponvory), siponimod (Mayzent), and ozanimod (Zeposia)) [14]	Lymphopenia, reactivation of viral infections (HSV and VZV), hemophagocytic syndrome, cryptococcal meningitis, reduced response to vaccinations, increased risk of basal cell carcinoma, cardiac conduction effects, macular edema, isolated skin tumors, HTN, and hypercholesterolemia
Ofatumumab (Kesimpta) [15]	Injection site reactions, infections, decreased immunoglobulin levels, and increased risk of malignancy
Cladribine (Mavenclad) [16]	Lymphopenia, herpes zoster, and increased risk of malignancy
Diroximel fumarate (Vumerity) [17]	Flushing, nausea, vomiting, abdominal pain, diarrhea, and increased risk of infections
Monomethyl fumarate [18]	Flushing, abdominal pain, nausea, diarrhea, flushing, and increased risk of infections

Current treatments versus established treatments: a comparison

In this section, several MS treatments will be compared, focusing on disease-modifying therapies and evaluating their efficacy based on relapse rates, disability progression, MRI measures, and quality of life outcomes. By analyzing these end points, we aim to provide a comprehensive overview of common MS treatments to assist patients and healthcare professionals in making informed decisions when having to choose between two or more common drugs. The literature review of research papers will specifically examine FDA-approved drugs such as interferons, glatiramer acetate, dimethyl fumarate, fingolimod, and teriflunomide and aims to concurrently identify the strengths, limitations, and suitability of different medications for various types of MS. Table 3 describes the current standard of treatment and relatively newer drug treatments that have been established for care.

Table 4 describes the category of medications used along with their trade names and their associated adverse effects. This is a particularly important point to be noted as the choice of medications in a patient’s treatment for relapsing-remitting multiple sclerosis can be hugely determined not only by the efficacy of the medications but also by the side effects that also correlate with the tolerance of side effects by the patients. Moreover, each patient requires an individualized treatment approach, and having a comprehensive overview of the adverse effects of medications aids in the successful treatment of patients.

S1P Modulators (Ponesimod, Siponimod, Fingolimod, and Ozanimod)

Mechanism of action: Selective and rapidly reversible S1P modulator causes the retention of lymphocytes in lymphoid organs. S1P modulators are metabolized by cytochrome P450 (CYP2C9 and CYP3A4); hence, drug interactions should be considered.

Adverse effects: Adverse effects include lymphopenia, reactivation of viral infections (HSV and VZV), hemophagocytic syndrome, cryptococcal meningitis, reduced response to vaccinations, increased risk of basal cell carcinoma, cardiac conduction effects, macular edema, isolated skin tumors, HTN, and hypercholesterolemia [14].

A systematic review conducted by Tong et al. was used to compare the efficacy of the S1P receptors in treating MS patients. A total of 13 randomized controlled studies were included, with 10,554 patients. A network meta-analysis (NMA) of S1P receptor modulators showed that fingolimod, laquinimod, siponimod, ozanimod, amiselimod, and ponesimod are superior in reducing the relapse rate compared to placebo in MS patients. The NMA indicated that amiselimod is the most effective treatment strategy as an S1P receptor modulator [19]. Although amiselimod currently is not an FDA-approved treatment for MS, studies thus far show promising results. The pharmaceutical company Mitsubishi Tanabe Pharma has conducted phase I and II (MOMENTUM study) trials, which looked at the effect of treatment with amiselimod at different dosages. It showed that 80% of patients treated with the drug’s highest doses for 48 weeks were free from lesions with active inflammation. Patients treated with amiselimod also had a 55% lower relapse rate compared to placebo [20].

Ponesimod Versus Teriflunomide

Ponesimod is a newly approved FDA drug used to treat relapsing forms of MS; it was approved based on phase III, double-blind, double-dummy, randomized clinical trial. The trial showed a better effect on activity markers than teriflunomide without additional safety issues. After a 24-week follow-up, phase II trials showed a mean lymphocyte reduction of 50%, 65%, and 69% in patients with RRMS randomized to ponesimod 10, 20, and 40 mg, respectively. After discontinuation, lymphocyte count returns to baseline values within one week (faster disease reactivation with ponesimod than other S1P modulators). Ponesimod has a selective effect on lymphocytes, showing a decrease in T and B cells in a dose-dependent pattern and no effect on natural killer cells [21].

Based on the results of the phase II trial, a phase III randomized controlled trial (RCT) was organized, in which the treatment efficacy was compared between ponesimod and teriflunomide. OPTIMUM RCT has split 1,133 patients into two groups in a one-to-one ratio; the first group (567 patients) had a 20 mg ponesimod once a day, and the second group (566 patients) had 14 mg teriflunomide once a day regimen for 108 weeks. The inclusion criteria for the trial were as follows: age 18-55 years, RRMS or SPMS with superimposed relapses, Expanded Disability Status Scale (EDSS) score between 0 and 5.5, and recent clinical or MRI disease activity. The trial showed that the relapse rate was significantly lower, with a relative reduction of 30.5%, for ponesimod versus teriflunomide. Ponesimod has also been shown to be more effective than teriflunomide in the reduction of combined unique active lesions (CUALs) per year (1.405 versus 3.164, relative reduction: 56%, p<0.001). The mean Fatigue Symptoms and Impacts Questionnaire (FSIQ) weekly symptoms score declined by 0.01 points and increased by 3.56 points in the ponesimod and teriflunomide arms, respectively (p=0.002). There was no difference noted in the proportion of patients with confirmed disability accumulation [21].

Fingolimod Versus Dimethyl Fumarate

A study compared the relapse rate, Expanded Disability Status Scale score, and MRI lesion metric in patients treated with fingolimod and dimethyl fumarate. A non-randomized comparison of the effect of the treatment was done. The eligibility criteria were as follows: patients enrolled in the Multiple Sclerosis Partners Advancing Technology and Health Solutions network and had follow-ups in the network for more than one year. In the overall cohort, there was no significant difference noted in the neuro performance or MRI outcomes, including the brain volume loss between DMF (702 patients) and FTY (600 patients) groups [22].

Ozanimod Versus Fingolimod

Comparative effectiveness assessment was conducted by matching adjusted indirect comparisons of the safety and efficacy of trial outcomes at first-dose cardiac monitoring. Ozanimod has been shown to be a better option as compared to fingolimod as it was associated with a lower risk of extended first-dose monitoring, conduction abnormalities, mean lymphocyte count reduction, and lower abnormal liver enzymes, herpetic infections, bradycardia [23].

Ozanimod Versus Dimethyl Fumarate

A matching-adjusted indirect comparison was used to compare the results of the clinical trials between ozanimod and dimethyl fumarate. The rate of confirmed disability progression at three months, annualized relapse rate (ARR), the proportion of patients relapsed, overall adverse effects, and discontinuations were significantly improved in patients treated with ozanimod. At six months, no difference was noted in confirmed disability progression between the two agents [24].

Ozanimod Versus Teriflunomide

A study was conducted that compared ozanimod and teriflunomide efficacy. The following parameters were considered: annualized relapse rate, the proportion of patients relapsed, confirmed disability progression (CDP) at three and six months, overall adverse effects, serious adverse effects, and discontinuations due to adverse effects. The ARR, overall AEs, serious AEs, and discontinuations due to adverse effects have improved in patients treated with ozanimod. It also showed significant improvement in CDP at three months, but not at six months, after the start of treatment compared to teriflunomide [25].

Fingolimod Versus Natalizumab

The REVEAL study is a one-year, randomized, rater-blinded and sponsor-blinded, prospective, head-to-head study comparing natalizumab and fingolimod in patients with active RRMS. This study showed a comparison of efficacy, relapse rates, benefits, and adverse effects between fingolimod and natalizumab. MS-naïve patients (patients who had not been treated before with DMT and had experienced ≥2 relapses in the previous year and ≥1 gadolinium (GD)-enhancing lesion on brain or spinal cord MRI scan) and patients who had been treated for six months with interferon or glatiramer acetate were included in the study. This study shows that both fingolimod and natalizumab have equal efficacy in treating MS, but a reduction in disease activity and relapses occurred more rapidly with natalizumab than with fingolimod [26].

Cladribine

Cladribine was associated with a significant 58% decrease in ARR versus placebo. It also was noted to be significantly better or the same as other DMT treatment alternatives. Cladribine was ranked fourth after alemtuzumab, natalizumab, and ocrelizumab. Cladribine showed significant improvement in CDP and no evidence of disease activity (NEDA) compared to placebo and showed no significant difference compared to other DMTs. Also, no significant difference was observed in adverse events risk compared to placebo and alternative DMTs. The research concluded that cladribine is an effective and safe alternative to other DMTs in active RRMS and high relapse activity (HRA) + disease activity on treatment (DAT) populations [27].

Diroximel Fumarate Versus Dimethyl Fumarate

Diroximel fumarate is a newly approved drug for treating relapsing forms of multiple sclerosis. After ingestion, it is converted into its active form, monomethyl fumarate (MMF). A study was conducted to evaluate the gastrointestinal tolerability of diroximel fumarate compared to dimethyl fumarate in relapsing-remitting MS patients. Statistically, shorter duration and lower intensity of gastrointestinal symptoms were observed in DRF-treated patients than in DMF. Also, DRF patients had a lower treatment discontinuation rate due to gastrointestinal adverse effects [17].

Monomethyl Fumarate Versus Dimethyl Fumarate

Monomethyl fumarate is a neuroprotective and immunomodulator that alters nuclear factor-like 2 transcription factor. It is the active metabolite of dimethyl fumarate and has a few side effects such as abdominal pain, nausea, diarrhea, and flushing. Monomethyl fumarate is metabolized via the tricarboxylic acid cycle without involving CYP 450 enzymes; this decreases drug interactions.

In a phase I comparative study between DMF and MMF on side effects and safety profile, 210 healthy volunteers were administered with equal molar weights of DMF and MMF twice a day for five weeks. The gastrointestinal tolerability profile of MMF assessed by the Modified Overall Gastrointestinal Symptom Scale (MOGISS) showed lower scores for vomiting and diarrhea with Bafiertam [28].

Ofatumumab Versus Teriflunomide

Ofatumumab is the first FDA-approved subcutaneous self-administered treatment option for patients suffering from RRMS. It is an anti-CD20 monoclonal antibody, which selectively depletes B cells. After receiving the initial dosing at week 0, 1, and 2, patients can subsequently receive maintenance therapy once monthly starting at week 4.

The ASCLEPIOS I and II clinical trials evaluated the relapse rate between ofatumumab and teriflunomide in patients suffering from RRMS. A total of 946 patients were assigned to receive ofatumumab, and 936 patients received teriflunomide for up to 30 months; the median follow-up was 1.6 years. The results of both these trials revealed that ofatumumab was associated with lower annualized relapse rates than teriflunomide [29].

The percentage of patients with disability worsening confirmed at three months was lower with ofatumumab (10.9%) than with teriflunomide (15%). The percentage of disability improvement confirmed at six months was higher with ofatumumab (11%) than with teriflunomide (8.1%). Additionally, the number of gadolinium-enhancing lesions per T1-weighted MRI scan, the annualized rate of lesions on T2-weighted MRI, and serum neurofilament light chain levels were all reduced with ofatumumab compared with teriflunomide. Notably, serious infections occurred more in patients who received ofatumumab (2.5%) compared with those who received teriflunomide (1.8%) [29].

In conclusion, the new treatments currently in the market for multiple sclerosis include S1P modulators such as ponesimod, siponimod, fingolimod, and ozanimod. These modulators have been shown to be effective in reducing the relapse rate and combined unique active lesions per year. Amiselimod, another S1P modulator, has been shown to be the most effective treatment strategy among the S1P receptor modulators. Ponesimod, a newly approved FDA drug, has been shown to have a better effect on activity markers than teriflunomide without additional safety issues. Ozanimod has been found to be a better option compared to fingolimod in terms of safety and efficacy. The new treatments have shown promising results and have demonstrated effectiveness in comparison to established treatments for multiple sclerosis.

Discussion

A Review of the Existing Guidelines

American Academy of Neurology: Multiple sclerosis, in its many forms, is a devastating chronic illness for many patients across the world. It is important for clinicians and researchers to understand the disease burden as well as the many facets of medications and their administration to patients. The current guidelines, as per the American Academy of Neurology (AAN), state that clinicians must counsel patients with MS about the severity of the illness and explain to them the available DMTs and their adverse effects, routes of administration, efficacy, and cost. Clinicians should not prescribe teriflunomide and cyclophosphamide to males with MS who have reproductive plans [30]. Clinicians can initiate alemtuzumab, fingolimod, and natalizumab in patients with highly active MS [30]. Azathioprine and cladribine can be prescribed in patients with RRMS who do not have access to approved DMTs. Physicians must elucidate that any discontinuation of DMTs can increase the relapse rates of MS. Switching DMT to another can be considered if a patient shows increased MRI lesion activity even after complete treatment adherence for a sufficient period. Injectables can be switched to non-injectables in patients who are intolerant to injections. In case of detecting new malignancies, patients on azathioprine, methotrexate, mycophenolate, cyclophosphamide, fingolimod, teriflunomide, alemtuzumab, or dimethyl fumarate are switched into another DMT [30].

European Academy of Neurology: According to the European Academy of Neurology (EAN), there is no current curative treatment for multiple sclerosis, and the pharmacological and therapeutic strategies are to reduce the risk of relapses as well as disability progression [31]. DMTs should be prescribed only in adequate infrastructure centers, which can provide proper monitoring of the patients and comprehensive assessment, as well as detection of side effects and capacity to address them immediately. Patients with active relapsing-remitting multiple sclerosis (RRMS) that have clinical relapses and/or MRI activity that is described by active lesions, contrast-enhancing lesions, and new or unequivocally enlarging T2 lesions assessed at least annually should be offered early treatment with DMTs [31]. In addition, with the wide range of current treatments available, patients who have active relapsing-remitting multiple sclerosis (RRMS) should choose drugs that will depend on the patient’s characteristics and comorbidities, disease severity or activity, drug safety profile, and accessibility of the drug [31]. It is also recommended to consult the summary of product characteristics (SPC) for the dosage, precautions for use and special warnings, contraindications, monitoring of side effects, and potential harms of the drugs [31].

In regard to the follow-up MRI, when monitoring treatment response in patients on DMTs, it is recommended to combine MRI with clinical measures to evaluate the disease evolution in the patients who are treated. A standardized reference brain MRI should be performed usually within six months of treatment onset, and it should be compared with a brain MRI performed 12 months after the treatment onset. The timing of both MRIs should be adjusted based on the drug’s mechanism of action (speed of action) and the patient’s disease activity; this includes the clinical and MRI measures [31]. New and enlarging T2 lesion measurement is the preferred MRI method, which is supplemented by gadolinium (GD)-enhancing lesions for monitoring treatment response. Standardized and high-quality MRI scans and interpretation by highly qualified readers with experience in multiple sclerosis (MS) are required in the evaluation of these parameters [31]. In treatment safety monitoring for patients on DMTs, standardized reference brain MRI should be performed annually in patients with low-risk progressive multifocal leukoencephalopathy (PML) and every 3-6 months in patients with high-risk PML and in high-risk PML patients who switch drugs at the onset where current treatment is discontinued and after which the new treatment is started [29]. Patients who are treated with interferon or glatiramer acetate who show evidence of disease activity should be offered a more efficacious drug, and on deciding which drug to switch to, while consulting the patient, patient characteristics and comorbidities, drug safety profile, and disease severity and activity should be considered. It is also recommended to consider starting another highly efficacious drug when treatment with a highly efficacious drug is stopped. The greater the disease activity, the higher the urgency to start new treatment; the half-life and biological activity of the previous drug as well as the potential for disease recurrence should all be taken into account when starting a new highly efficacious drug. When making these treatment decisions, the possibility of disease recurrence or rebound when stopping treatment (particularly with natalizumab) should be considered [31].

When it comes to long-term treatment, the European Academy of Neurology (EAN) recommends that all women of childbearing potential should be advised that DMTs are not licensed during pregnancy, except for glatiramer acetate with a dose of 20 mg/mL. For those women who are planning pregnancy, using interferon or glatiramer acetate should be considered if there is a high risk of reactivation of the disease until the pregnancy is confirmed. Continuing this treatment during pregnancy should also be considered in some active or very specific cases. In addition, it is advised to delay pregnancy for those women with persistent high disease activity. However, for those who still decide on becoming pregnant or have an unplanned pregnancy, it is advised that the patient may consider treatment with natalizumab throughout the pregnancy after knowing the potential implications and an alternative therapeutic option such as treatment with alemtuzumab for planned pregnancy with very active cases, given that the patient strictly observes a four-month interval from the latest infusion until conception [31].

National Institute for Health and Care Excellence Guidelines (NICE): The current National Institute for Health and Care Excellence (NICE) guidelines state that peginterferon beta-1a is recommended for adult patients with relapsing-remitting multiple sclerosis; it showed that this drug slows the progression of the disease and reduce the frequency of relapses. It is also a cost-effective drug treatment for relapsing-remitting multiple sclerosis (RRMS) [32]. Ozanimod is not recommended for patients with relapsing-remitting multiple sclerosis (RRMS) due to its unclear progression of disability; however, it reduces the number of relapses and brain lesions [33]. According to the NICE guidelines, cladribine is the recommended therapy for adults who have highly active multiple sclerosis in consideration that the patient has rapidly evolving severe relapsing-remitting multiple sclerosis (RRMS) who had at least two relapses in the previous year and one T1 gadolinium-enhancing lesion at baseline MRI or significant increase in T2 lesion load compared to the previous MRI or who has not responded to treatment using disease-modifying therapy, which is defined as one relapse in the previous year and MRI evidence of disease activity [34]. For adult patients with relapsing-remitting multiple sclerosis (RRMS) that is contraindicated or not suitable to alemtuzumab, ocrelizumab is recommended as an alternative treatment [35]. When it comes to first-line treatments for relapsing-remitting multiple sclerosis (RRMS), NICE guidelines have stated that interferon beta-1a, interferon beta-1b (Extavia), and glatiramer acetate are recommended, with considerations for interferon beta-1b (Extavia) that the patient has had two or more relapses within the last two years. There is one exception: interferon beta-1b (Betaferon) is not recommended due to its inconvenience of usage, and its cost-effectiveness is not at par with what the National Institute for Health and Care Excellence (NICE) and National Health Service (NHS) consider as a cost-effective drug [36].

The current different guidelines have different approaches when it comes to the treatment of multiple sclerosis. The AAN specifically stated patient adherence or compliance, as well as the switching of therapies or treatments, but not the EAN guidelines. However, the EAN guidelines particularly mentioned the implementation of the establishment of multiple sclerosis centers for better patient assessment and monitoring, as well as detection and management of adverse effects. Also, the usage of MRI is discussed more in detail by the EAN guidelines than by the AAN and NICE guidelines [37].

Only the AAN guidelines stated that teriflunomide and cyclophosphamide should be considered in males who have multiple sclerosis and have reproductive plans due to their possible risks. Both AAN and EAN guidelines advise not to use DMTs in patients during pregnancy and who are planning to conceive; however, the EAN considers the continuation of interferon beta and glatiramer acetate until pregnancy is confirmed and for those who are planning to conceive as well as considering to still continue treatment during the pregnancy if the patient is at risk for multiple sclerosis relapse or reactivation [37].

In comparison with the three said guidelines, only in the NICE guideline recommendations, all drugs include the condition that the companies provide it according to commercial arrangements (in which the patients should contact pharmaceutical companies to gain simple discount patient access schemes), and all the drugs that this guideline recommend are cost-effective drugs.

The guidelines for MS management are similar across the American Academy of Neurology (AAN), European Academy of Neurology (EAN), and NICE guidelines. The goal of all management strategies is to reduce the risk of relapses and focus on reducing the progression of disability by administering DMTs. The EAN recommends prescribing DMTs based on the patient’s characteristics, comorbidities, disease severity, drug safety profile, and accessibility, while the AAN recommends a precise list of medications (alemtuzumab, fingolimod, and natalizumab) for highly active MS as a primary therapeutic approach. All three guidelines share some similarities in terms of counseling patients on DMTs and monitoring treatment response; they differ in the choice of drugs and the frequency of MRI scans. AAN guidelines focus on the initiation and switching of DMTs and provide recommendations for specific drugs. EAN guidelines focus on early treatment with DMTs, the importance of follow-up MRI, and recommendations for choosing drugs. NICE guidelines provide recommendations for specific DMTs and the importance of extensive regimen review after two years of treatment.

Different Approaches to Therapy

According to La Mantia et al., a comparison between interferon beta and glatiramer acetate showed similar benefits and risks. However, there is a significantly lower relapse ratio at 36 months with glatiramer acetate than with interferon beta. Treatment discontinuation due to adverse effects was the same with both therapies [38]. Teriflunomide and dimethyl fumarate are oral drugs among first-line treatments for MS, while interferon beta and glatiramer acetate are injectables. According to Vermersch et al., compliance was higher with teriflunomide than with other first-line drugs such as interferon beta, glatiramer acetate, and dimethyl fumarate [39]. According to Zhang et al., the reasons cited by patients for discontinuing teriflunomide were persistent disease activity despite taking treatment (37.3%), poor tolerability (24%), inability to afford teriflunomide medication (17.3%), and failure in obtaining treatment during COVID-19 (13.3%). The adverse drug effects reported with teriflunomide use are hair thinning, increased liver enzymes, leukopenia, diarrhea, skin rash, and weight loss [40]. The treatment persistence of dimethyl fumarate was lower than fingolimod, although both have comparable efficacy. Dimethyl fumarate showed a reduction in ARR by 29% compared with glatiramer acetate. Treatment discontinuation during the first year of initiation is more frequent with dimethyl fumarate than with fingolimod due to its lack of tolerability [41]. According to Yang et al., the adverse effects of fingolimod are bradycardia, infections, altered liver function test (LFT), eye events, back pain, and muscle spasms. The study stated that the adverse effects of fingolimod are manageable, and treatment discontinuation due to adverse effects is less [42]. According to Butzkueven et al., reduction in disease activity and relapses occurred more rapidly with natalizumab than with fingolimod, although both have equal efficacy in treating MS [26]. According to Rodríguez de Castro et al., patients on natalizumab developed severe adverse effects such as PML, other opportunistic infections, malignancies, pulmonary emboli, and hepatotoxicity [43]. However, this study has concluded, thus far, that no confirmed cases of PML have been reported, which supports existing literature that the occurrence of PML is very rare, which takes into consideration that the risk-benefit ratio favors the drug as having more benefits than risks. The rate of adverse effects, however, was reported to be at 50%. On comparing the benefits and risks of first-line and second-line therapies of MS, we find that second-line drugs show greater efficacy than first-line drugs but are frequently discontinued due to their severe adverse effects.

MS, like any other disease, does not have a predictable progression of disease over time. Some patients may not be as impacted as other patients over the years. So, it is highly unpredictable to say when to start the treatment, but early treatment would be suggestible. Untreated patients or patients who are off DMTs may progress to SPMS. So, it is important to reduce relapses and increase the interval between relapses because the increased frequency of relapse leads to an increase in the progression of the disease and an increase in the number of brain lesions on MRI. There is autoimmune destruction happening in the brain of multiple sclerosis (MS) patients. Thus, delaying treatment will only increase relapses and worsen the condition forever. Relapses also affect the patient’s day-to-day activities, so it is important to get treatment as soon as possible.

For any patient to be compliant with treatment, the drug should be administered orally, the time interval between two dosages should be more, and it should be cost-effective and should cause fewer adverse effects. Intolerability of injectables and severe adverse effects have been the reasons for patients to discontinue the treatment. Thus, guidelines should focus on these aspects as well.

Table 5 compares the different variables taken into consideration when deciding which line of therapy to use for a patient with RRMS. As we shift from first-line treatment to second-line treatment, the adverse effects increase, along with efficacy, which restricts clinicians to only use them in progressed cases. Many studies out there are more focused on efficacy, but not much research is done on the adverse reactions of DMTs. Thus, studies should be carried out to document the severe adverse effects of all therapeutic options for multiple sclerosis.

**Table 5:** Comparison of first-line and second-line therapies for RRMS RRMS: relapsing-remitting multiple sclerosis

Criteria	First-line therapies	Second-line therapies
Efficacy	Moderate to high	High
Compliance	Low to moderate	Moderate to high
Relapse rate	Moderate	Low
Adverse effects	Mild to moderate	High
Discontinuation rate	Moderate	High
Cost	Moderate to high	High

## Conclusions

This literature review has undertaken a comprehensive examination of therapeutic options for multiple sclerosis, serving as a valuable resource for healthcare practitioners when choosing a treatment option and the research community when advancing therapeutic options. The available disease-modifying therapies (DMTs) for relapsing-remitting MS (RRMS) are classified into first-line, second-line, and third-line treatments and further classified based on their clinical end points such as safety, efficacy, and tolerability. The newer DMTs for RRMS were also discussed and compared to older therapies based on several important factors. Different guidelines for MS were examined and discussed in this paper to allow for a quick comparison when choosing a school of thought to approach MS. Ultimately, the different guidelines for MS management all recommend administering DMDs to reduce the risk of relapses and disability progression, with the focus on patient characteristics, disease severity, and accessibility. Early treatment is essential to prevent disease progression and improve the quality of life of MS patients. More research is needed to compare the adverse effects of DMTs, develop cost-effective drugs, and improve patient compliance. Ultimately, advancements in MS treatment can potentially change a patient’s disease course and improve the standard of care.
